# Davemaoite as the mantle mineral with the highest melting temperature

**DOI:** 10.1126/sciadv.adj2660

**Published:** 2023-12-06

**Authors:** Kun Yin, Anatoly B. Belonoshko, Yonghui Li, Xiancai Lu

**Affiliations:** ^1^ Research Center for Planetary Science, College of Earth Sciences, Chengdu University of Technology, Chengdu 610059, China.; ^2^ Frontiers Science Center for Critical Earth Material Cycling, School of Earth Sciences and Engineering, Nanjing University, Nanjing 210023, China.; ^3^ Condensed Matter Theory, Department of Physics, AlbaNova University Center, Royal Institute of Technology (KTH), 10691 Stockholm, Sweden.; ^4^ National Research University Higher School of Economics, 123458 Moscow, Russia.; ^5^ Department of Physics, University of South Florida, Tampa, FL 33620, USA.; ^6^ National Supercomputing Center in Chengdu, Chengdu 610299, China.; ^7^ State Key Laboratory for Mineral Deposit Research, School of Earth Sciences and Engineering, Nanjing University, Nanjing 210023, China.

## Abstract

Knowledge of high-pressure melting curves of silicate minerals is critical for modeling the thermal-chemical evolution of rocky planets. However, the melting temperature of davemaoite, the third most abundant mineral in Earth’s lower mantle, is still controversial. Here, we investigate the melting curves of two minerals, MgSiO_3_ bridgmanite and CaSiO_3_ davemaoite, under their stability field in the mantle by performing first-principles molecular dynamics simulations based on the density functional theory. The melting curve of bridgmanite is in excellent agreement with previous studies, confirming a general consensus on its melting temperature. However, we predict a much higher melting curve of davemaoite than almost all previous estimates. Melting temperature of davemaoite at the pressure of core-mantle boundary (~136 gigapascals) is about 7700(150) K, which is approximately 2000 K higher than that of bridgmanite. The ultrarefractory nature of davemaoite is critical to reconsider many models in the deep planetary interior, for instance, solidification of early magma ocean and geodynamical behavior of mantle rocks.

## Teaser

Computer simulations predict the third most abundant mineral in Earth's lower mantle is likely the most refractory one.

## INTRODUCTION

Melting temperature of minerals in the deep rocky interior of Earth provides essential constraints on many key thermal-chemical processes during the planetary evolution, such as crystallization of early magma ocean (*1*), origin of thermochemical anomalies (*2*), and fate of recycled basaltic crust (*3*). The Earth’s lower mantle, ranging from depth of 660 to 2890 km below the surface, is mainly composed of three kinds of minerals, viz., bridgmanite, ferropericlase, and davemaoite. Here, we investigated the melting of two minerals, bridgmanite and davemaoite in MgSiO_3_ and CaSiO_3_ composition, respectively. The melting curve of the MgSiO_3_ system is basically known with little controversy between various measuring and computational methods (*4*–*8*), so we calculated this system as a control group. At present, melting temperatures in the CaSiO_3_ system have only been measured up to ~55 GPa (*7*, *9*, *10*), whereas a large portion of the lower mantle has not yet been explored by experiments. Moreover, the available laser-heated diamond anvil cell (LHDAC) experimental results have strong disagreement with each other (*7*, *10*) and obviously contradict with the low-pressure multi-anvil measurement (*9*). Besides experiments, a few theoretical studies have obtained the melting curves of davemaoite under the lower mantle conditions (*11*–*13*). However, discrepancies between these computational studies are also prominent, for example, the melting points at the pressure of core-mantle boundary (CMB) differ as large as 1400 K (*11*, *13*). As an important constituent in the lower mantle, a well-defined melting curve of davemaoite is crucial for building a self-consistent thermodynamic model of the deep Earth (*14*, *15*). Evidently, it is an essential need to investigate this melting problem again to reconcile the currently controversial results among various experimental and computational studies.

At ambient conditions, calcium silicate CaSiO_3_ is stable in the form of wollastonite. After a series of solid-solid phase transitions underwent at pressures below ~13 GPa, it transforms into a perovskite phase (*9*, *16*, *17*). At low temperature (below ~1000 K), the perovskite phase has a tetragonal structure. At elevated temperature, it is stabilized in a cubic structure with space group $Pm\bar{3}m$ (*18*–*20*). Because the kinetic barrier of back conversion is small, the cubic perovskite is easily transformed to low-pressure polymorphs after pressure release; therefore, it is hardly seen at Earth’s surface (*21*, *22*). Recently, a rare sample of this phase was found in diamond inclusion and named davemaoite in honor of high-pressure geoscientist D. (Ho-kwang) Mao (*23*). Although disputes about this natural presence still exist (*24*), based on mineral physics experiments, davemaoite is believed to remain stable in a wide range of pressures and temperatures in the lower mantle (*25*, *26*). Davemaoite was well known as a geochemically important phase due to its capability to accommodate large-ion incompatible elements (*27*–*29*). Recently, many distinct physical properties of this phase have been revealed, such as low shear modulus (*30*, *31*), high thermal conductivity (*32*), and weak rheological strength (*33*). In this study, we will show another special property of davemaoite, that is, the ultrarefractory nature at high pressure.

## RESULTS

### Melting curves of MgSiO_3_ and CaSiO_3_

In this work, we obtained the melting curve of davemaoite between 13 and 136 GPa, covering its stability field in the mantle. As a comparison, we also obtained the melting curve of MgSiO_3_ bridgmanite at pressures covering the lower mantle. We determined their melting curves using a purely theoretical approach based on first-principles molecular dynamics (FPMD) simulations. Determination of the melting curve requires to sample many temperature points on the curve at different pressures and then fit these points to an empirical equation. These sampled points can be obtained directly from calculation of melting point explicitly, but this way requires too much computational effort and usually produces less-smooth curve because of large uncertainties in the melting point. Here, we only use one melting point on the curve as the reference point. Then, we derive the whole melting curve by constructing the volume-entropy-energy surface, the so-called Gibbs thermodynamic surface, based on the predefined reference point (*34*). This way achieves similar goal as the integration of Clausius-Clapeyron equation (*5*). Details of these methods and relevant parameters in the simulations are given in Materials and Methods.

We have obtained the equilibrium melting point (*T*_m_) using the Z method (*11*, *35*). We have tried to use different reference melting points to derive the melting curve (table S1). We find that using different reference points provide almost identical melting curves lying within each other’s uncertainty bounds (fig. S1). Here, we report the best fit of the melting curve represented by the following Kechin equation (*36*)

$$T_{m}(P)=T_{0}{\left(1+\frac{P-P_{0}}{a}\right)}^{b}\exp\left(-\frac{P-P_{0}}{c}\right)$$ (1)

Parameters of the above equation for the melting curve of MgSiO_3_ bridgmanite are as follows: *T*_0_ = 2900 K, *P*_0_ = 22.4 GPa, *a* = 15.6 GPa, *b* = 0.364, and *c* = 1066 GPa. Its melting temperatures change from ~3050(50) K at 25 GPa to ~5600(200) K at the pressure of the bottom of lower mantle (~136 GPa). Parameters of the above equation for davemaoite are as follows: *T*_0_ = 4020 K, *P*_0_ = 24.6 GPa, *a* = 14.9 GPa, *b* = 0.324, and *c* = 2610 GPa. Melting temperatures of davemaoite change from ~2480(80) K at 13 GPa to ~7700(150) K at 136 GPa. The upper and lower uncertainty bounds of melting curves are constrained by changing the reference point on the curve randomly within its SEs (table S2).

### Comparison with previous theoretical calculations

**Fig. 1.**
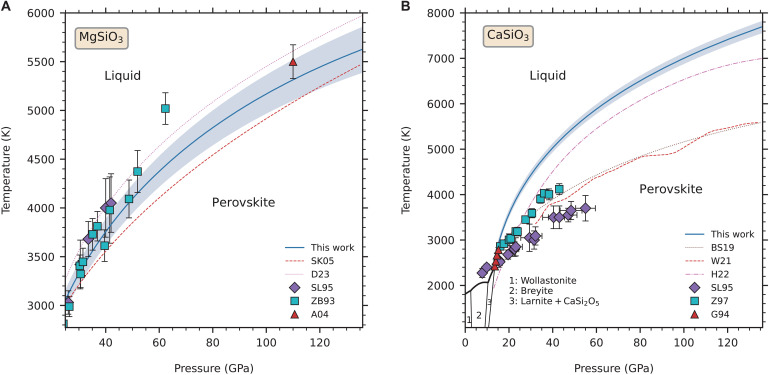
Melting curve of two perovskites under high pressures up to the core-mantle boundary. Melting curve is shown as solid blue curve. Shaded area around the melting curve indicates the upper and lower uncertainty bounds of the curve. (**A**) Melting of MgSiO_3_ bridgmanite. LHDAC experimental data: SL95, Shen and Lazor (1995) (*7*) and ZB93, Zerr and Boehler (1993) (*8*); Shock-wave experimental data: A04, Akins *et al.* (2004) (*6*); and previous computational data based on ab initio calculations: SK05, Stixrude and Karki (2005) (*5*); D23, Deng *et al.* (2023) (*4*). (**B**) Melting of davemaoite. Multi-anvil experimental data: G94, Gasparik *et al.* (1994) (*9*); LHDAC experimental data: SL95, Shen and Lazor (1995) (*7*) and Z97, Zerr *et al.* (1997) (*10*); and previous computational data based on ab initio calculations: BS19, Braithwaite and Stixrude (2019) (*11*); WS21, Wilson and Stixrude (2021) (*12*); and H22, Hernandez *et al.* (2022) (*13*). Black lines display the phase transition boundaries of solid phases below 13 GPa (*9*, *16*, *17*).

The melting curves of MgSiO_3_ bridgmanite have been reported by two FPMD simulations based on density functional theory (DFT) previously. Stixrude and Karki (*5*) first predicted the melting curve by integrating the Clausius-Clapeyron equation. In their study, the integration constant is set by assuming a reference point the melting point at 25 GPa measured by experiment. Very recently, Deng *et al.* (*4*) determined the melting curve by performing two-phase simulations using a machine learning potential trained from FPMD calculations. In the present study, we derived the melting curve of MgSiO_3_ bridgmanite using a reference point obtained by the Z method, that is, *P* = 89.1(1.3) GPa and *T* = 4989(188) K. Figure 1A shows that our melting curve lies between the two previous theoretical studies. The upper and lower uncertainty bounds of our melting curve are in general agreement with previous estimations.

Unlike the MgSiO_3_ bridgmanite, melting curves of davemaoite obtained based on DFT calculations display large discrepancies (*11*–*13*). Figure 1B indicates that our predicted melting curve of davemaoite is the highest one among all computational studies. To assess the discrepancies between different theoretical works, we have purposely chosen the same constant volume (*V* = 5.80 cm^3^/mol per atom) in the Z method as previous study of Braithwaite and Stixrude (*11*). We followed the same waiting time analysis procedure and adopted the PBEsol functional (*37*) as did by pervious study, except that we used a smaller timestep. The melting point we obtained at this constant volume is *P* = 48(1) GPa and *T* = 5425(150) K. However, the melting point obtained by Braithwaite and Stixrude (*11*) at the same volume is *P* = 44.2(2) GPa and *T* = 4020(60) K, which is much lower than that of ours (fig. S1). During the Z method simulations, we observed a drift in the internal energy, which should be conserved in the microcanonical (NVE; *N*-number of atoms, *V*-volume, *E*-energy) ensemble simulations, is unavoidable. After testing different settings in the simulations, we noticed timestep is one of the most important factors that affect the energy conservation. Therefore, we used a smaller timestep (0.5 fs) in our simulations. This setting makes the energy drift of internal energy become less than 0.2 meV/atom per ps for a length of 30 ps (fig. S2). Using a larger timestep (e.g., 1 fs) makes the energy drift an order of magnitude larger than ours; thus, we speculate that the underestimation of melting point in previous work is perhaps due to the large accumulated energy drift in the NVE ensemble simulations.

Wilson and Stixrude (*12*) determined the melting curve by 2PT (two-phase thermodynamic) free-energy method, and their results are in support of the study of Braithwaite and Stixrude (*11*). They derived the free energies by calculating absolute entropies using the vibrational density of states, which is obtained from Fourier transformation of the velocity autocorrelation function (*38*). Very recently, Hernandez *et al.* (*13*) repeated the 2PT free-energy calculations. In addition to 2PT method, they also calculated the free energies using thermodynamic integration method (*39*). They found the inconsistency of melting curves between Wilson and Stixrude (*12*) and theirs mainly results from the overestimation of liquid entropies obtained from the 2PT method. We have repeated the thermodynamic integration method calculations by ourselves and found good agreement with the results of Hernandez *et al.* (*13*) (table S3). According to former experience of using 2PT method, entropy of liquid phase calculated by this method is strongly affected by the way of how to estimate the partial molar volume of each atomic species in a polyatomic system (*40*). Wilson and Stixrude (*12*) adopted the simple one-fluid approximation, in which all partial molar volumes of different atomic species are regarded equal. According to Hernandez *et al.*(*13*), the deviation of melting point calculated by the 2PT method and thermodynamic integration method enlarges with increasing pressure; therefore, we speculate that the 2PT method within the framework of one-fluid approximation is not accurate enough for calculation of liquid entropy at high pressures, especially for CaSiO_3_, a polyatomic system with dissimilar size of species.

Although Hernandez *et al.* (*13*) predicted much higher values of temperature than previous calculations (*11*, *12*), their melting data are still ~500 K lower than that of our predictions on average. To reconcile this discrepancy, we carefully investigate the procedure of their calculations. We find that if we consider the effect of pressure correction caused by different exchange-correlation approximations in DFT calculations, the agreement of equation of state between calculation and experiment can be greatly improved (see Materials and Methods and fig. S3). Therefore, we repeated the melting curve calculations using the Perdew-Burke-Ernzerhof (PBE) functional (*41*) as what was used in (*13*), previously. The authors of (*13*) have adopted three different methods to constrain the melting curve, i.e., small-cell coexistence (SCC) method, large-cell coexistence (LCC) method, and thermodynamic integration method. After changing to the PBE functional, we find that good agreement was made between our melting curve and the results of all the three methods, especially in excellent agreement with the SCC method (fig. S4). Small discrepancy still exists at pressures higher than 80 GPa for the thermodynamic integration method. The accuracy of free energies calculated by thermodynamic integration method is sensitive to the number of λ parameter, a coupling parameter that connects the reference system and the target system, in the numerical quadrature. However, at high pressures, λ parameters were not sampled enough in the work of Hernandez *et al.* (*13*) because DFT calculations are hard to converge with small λ values. Thus, we suspect that the remaining discrepancy is probably caused by the large uncertainties in thermodynamic integration method at high pressures due to insufficiently sampled λ points at smaller values (fig. S5).

### Comparison with previous experimental measurements

Melting temperatures of MgSiO_3_ bridgmanite have been measured by static-compression experiments based on LHDAC technique at pressures lower than 60 GPa (*7*, *8*) and by shock-wave experiment at one high-pressure point more than 100 GPa (*6*). Most of these data points agree well with the theoretical predictions within their uncertainties. So, we can say that the melting curve of MgSiO_3_ bridgmanite has generally achieved a consensus between various experimental and computational studies (Fig. 1A). For davemaoite, melting temperatures have been measured by multi-anvil (*9*) and LHDAC techniques (*7*, *10*). At low pressures (13 to 16 GPa), the melting temperatures of multi-anvil and LHDAC experiments are mutually consistent. The main discrepancy between them within this pressure range is the slope of melting curve. Multi-anvil results indicated a very steep initial slope (20 K/kbar); however LHDAC results hinted much lower slopes (4 to 6 K/kbar). In previous experiments on davemaoite, the multi-anvil measurement was performed by resistance heating and thermocouple technique (*9*), whereas the LHDAC measurement was performed by laser heating and thermal emission spectrum fitting technique (*7*, *10*). The former technique generally yields smaller uncertainties compared to the latter. Our calculation is in excellent agreement with the multi-anvil results and the LHDAC data at low pressures (Fig. 1B). The predicted melting curve supports the steep slope and coincides with the extension of solid-solid transition boundary between larnite (Ca_2_SiO_4_) + CaSi_2_O_5_ and davemaoite. At higher pressures (>16 GPa), melting temperatures of the two LHDAC experiments are contradictory (*7*, *10*). Both of them are substantially lower than our predicted melting temperatures. The disagreement between the two experiments is perhaps because they adopted different laser heating techniques, which is YAG heating versus CO_2_ heating. In YAG heating, rhenium was used to absorb the radiation, where the chemical reaction between rhenium and CaSiO_3_ may be the cause of the underestimation of melting temperatures in (*7*). It is hard to say why study of (*10*), which used improved laser heating technique, still obtained such low temperatures. Here, we only raise one possibility for reference. In both LHDAC experiments, the plateau in the plot of laser power versus temperature has been used as the criterion to identify the onset of melting. It has been found that this criterion might be problematic for the case of periclase (MgO) because with increasing pressure multiple temperature plateaus exist in this kind of plot for periclase (*42*). Therefore, if there also exists such phenomenon for davemaoite, viz., the true melting takes place at the second plateau that has a higher temperature, then misusing the first plateau would result in an underestimation of melting temperatures. Because melting temperature of davemaoite is too high, the second plateau perhaps was not yet reached in previous experiments.

### Verifying melting temperature of CaSiO_3_ by large-scale two-phase simulations

**Fig. 2.**
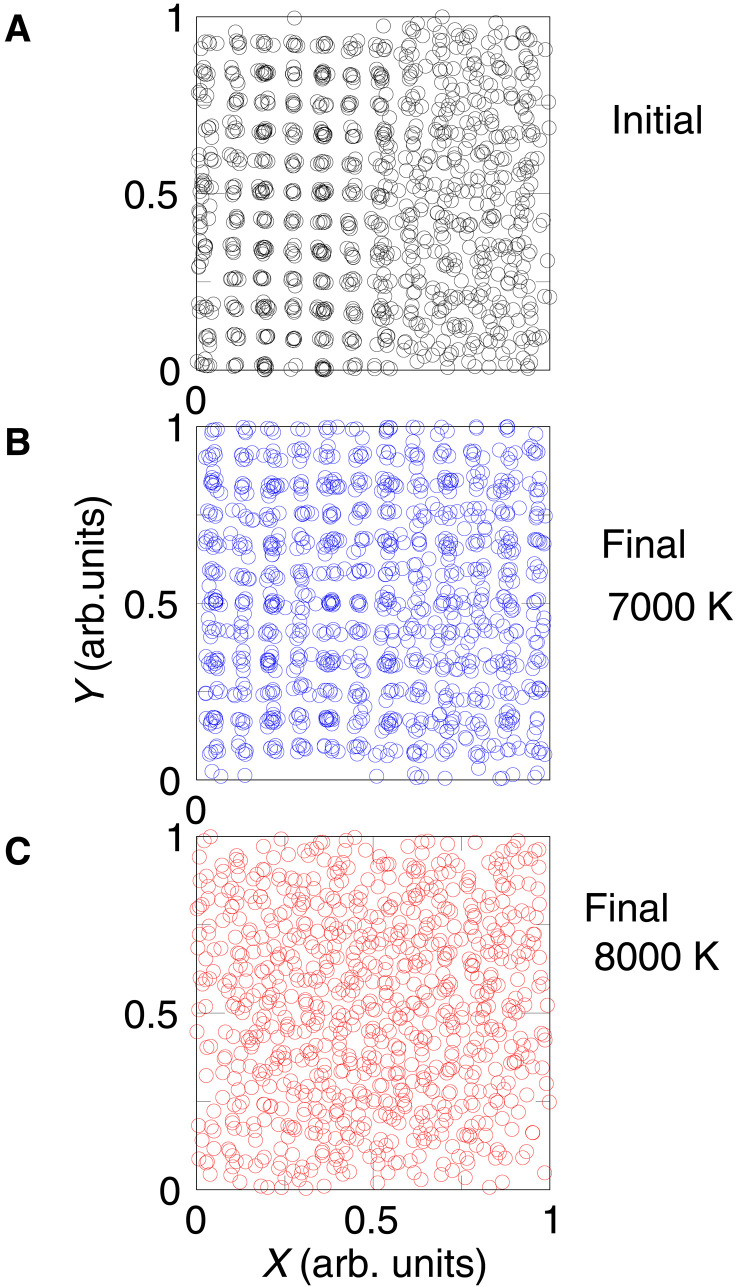
*XY* projections of positions of 1080 atoms of the CaSiO_3_ system in the computational cells of the two-phase method. (**A**) The initial structure where the solid part is on the left-hand side and the liquid part is on the right-hand side. (**B**) The final configuration after approximately 50,000 steps of FPMD simulation at *P* = 150 GPa and *T* = 7000 K. (**C**) The final configuration after approximately 20,000 timesteps and a temperature of 8000 K. The simulation at 7000 K (middle) leads to freezing, while the simulation at 8000 K (bottom) leads to melting.

To further corroborate our results, we also performed two-phase method calculations under an isobaric-isothermal (NPT) ensemble. The two-phase method (*43*) is often confused with coexistence method (*44*). For example, the so-called LCC method, which has been adopted in the previous work (*13*), belongs to the coexistence method, not the two-phase method. Both two-phase method and coexistence method start from a cell consisting of liquid and solid parts with a common interface (Fig. 2A). The unique feature of the two-phase method is that the cell becomes homogeneous after equilibration, whereas in the coexistence method the common interface must always persist. This feature of two-phase method is essentially advantageous in performing large-scale FPMD simulations. To run a FPMD simulation with more than a thousand atoms for tens of thousands of steps would require enormous computational effort. From an operational point of view, homogenization is much easier to identify than a persistent interface. One does not have to run it to completely homogeneous state—the trend becomes pretty obvious by simply looking at the change of the radial distribution function (RDF). The two-phase simulation runs at *P* = 150 GPa, and temperatures of *T* = 7000 K and lower than 7000 K resulted in the solid structure. The run at *T* = 7000 K required 50,000 timesteps, yet we observed some traces of liquid that remained in the cell (Fig. 2B), although part of the liquid had crystallized. This low crystallization is likely due to two reasons. First, the temperature is likely very close to the melting temperature, and second, the complicated structure makes the kinetics of crystallization slow. At *T* = 8000 K, the crystal melts. The melting proceeds rather quickly, and after 20,000 timesteps, we see a homogeneous liquid (Fig. 2C). The RDF figure corroborates this (fig. S6). We see that as the simulation progresses, the RDF averaged over the 5000 timesteps becomes more structured for the run at *T* = 7000 K and less structured at *T* = 8000 K. The run at 7000 K took approximately 8 weeks of calculations using 512 cores, so we hesitated to make the temperature interval much closer. For our purpose, such a large interval was sufficient to get convinced that the melting temperature of davemaoite is much higher than that was estimated before. We also performed the two-phase method calculations using machine learning force field (MLFF), which was trained using on-the-fly FPMD data at the CMB pressure. This MLFF potential allows us to run longer simulation time (e.g., 100,000 timesteps. At *T* = 7500 K, we observed a completely crystallization process of the two-phase simulation) (movie S1). The result of MLFF potential is in full agreement with the pure FPMD result, which suggests that the melting of davemaoite at the CMB pressure is confined between temperatures 7000 and 8000 K.

### Properties along melting curves

**Fig. 3.**
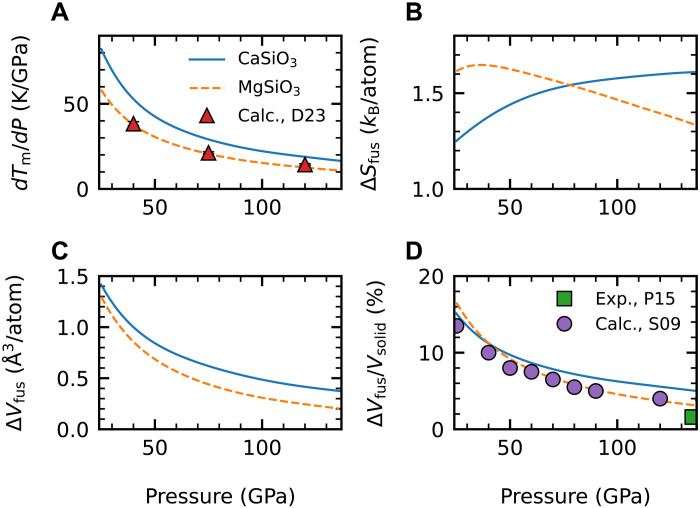
Computed properties of melting for two perovskites under pressures between 25 and 136 GPa. CaSiO_3_ davemaoite (solid line) and MgSiO_3_ bridgmanite (dashed line). (**A**) The Clapeyron slope. (**B**) Entropy of fusion. (**C**) Volume of fusion. (**D**) Ratio between volume of fusion and volume of the solid phase in percentage. Previous results of calculations (Calc.) and experiments (Exp.) for MgSiO_3_ bridgmanite are marked as symbols: D23, Deng *et al.* (2023) (*4*); S09, Stixrude *et al.* (2009) (*46*); and P15, Petitgirard *et al.* (2015) (*47*).

The Clapeyron slope is one of the most important properties along the melting curve because it predicts the rate of increase in melting point per unit increase in pressure. This quantity can be estimated by the following equation, $\frac{dT_{m}}{dP}=\frac{\text{Δ}V_{\mathrm{fus}}}{\text{Δ}S_{\mathrm{fus}}}$, where ∆*V*_fus_ = *V*_liquid_ − *V*_solid_ and ∆*S*_fus_ = *S*_liquid_ − *S*_solid_ are the volume and entropy of fusion between liquid and solid when melting occurs, respectively. We have shown the Clapeyron slopes and related properties in Fig. 3. The Clapeyron slopes of MgSiO_3_ bridgmanite are in very good agreement with a recent theoretical study using machine learning–based ab initio calculations (Fig. 3A) (*4*). The Clapeyron slopes of davemaoite are always higher than that of MgSiO_3_ bridgmanite up to the CMB pressure. For the entropy of fusion, a crossover occurs at moderate lower-mantle pressure (Fig. 3B). The volume of fusion is close between the two lower-mantle minerals at low pressures but becomes increasingly larger as pressure increases (Fig. 3C). Therefore, the high melting slopes of davemaoite are mainly caused by the small entropies of fusion of davemaoite at lower pressures and the more rapid decrease in the volume of fusion of bridgmanite at higher pressures. The smaller entropy of fusion of davemaoite possibly results from the large entropy of the solid phase contributing from the fast tilt switches of cation-oxygen octahedra at high temperatures in the cubic-structured perovskites (*45*). Last, the relative changes in volume of fusion with respect to the volume of the solid phase of MgSiO_3_ bridgmanite are in good agreement with previous theoretical (*46*) and experimental (*47*) estimates (Fig. 3D).

### Relative viscosity contrast between MgSiO_3_ and CaSiO_3_

**Fig. 4.**
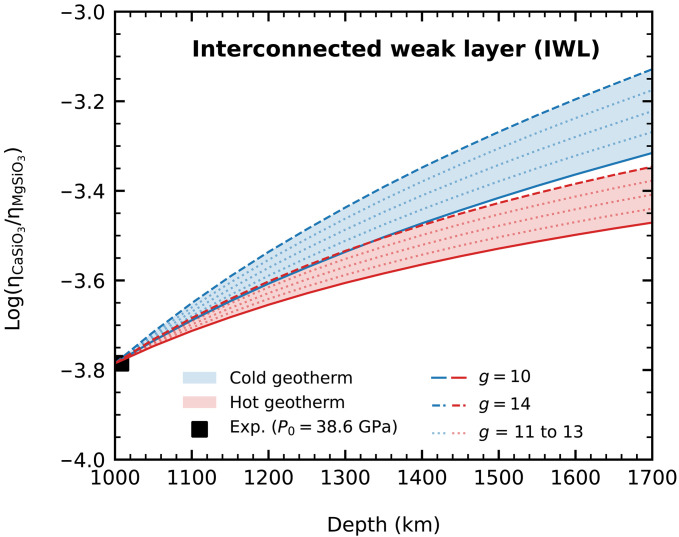
Depth-dependent profile of viscosity contrast between CaSiO_3_ and MgSiO_3_ perovskites in the deep Earth. The viscosity contrast was calculated under an IWL scenario. This scenario assumes a uniform stress progressed deformation that has led to the development of the interconnected layer of the weaker phase. Two mantle geotherms, the “cold geotherm”(*50*) and “hot geotherm”(*51*), were considered in the modelling. Models were built with five different values of homologous temperature scaling factor *g* between 10 and 14 and a reference value of viscosity ratio at pressure *P_0_* = 38.6 GPa from the experiment (*33*).

Following a homologous temperature scaling method (*48*), the viscosity of a mineral at high pressure *P* over its viscosity at a reference state with pressure *P*_0_ is given by

$$\frac{\eta (P)}{\eta (P_{0})}=\frac{T(P)}{T(P_{0})}\frac{\text{Ω}(P_{0})}{\text{Ω}(P)}\exp\left\{g\left[\frac{T_{m}(P)}{T(P)}-\frac{T_{m}(P_{0})}{T(P_{0})}\right]\right\}$$ (2)

where η, Ω, *T*, *T*_m_, and *g* are viscosity, molar volume, geotherm temperature, melting temperature, and a dimensionless scaling factor, respectively. If we know the viscosity contrast between two minerals at a lower reference pressure, then we can estimate their viscosity contrast at a wide range of depths using the melting curves of these minerals by the above scaling model. Recently, Immoor *et al.* (*33*) measured the plastic strength of davemaoite under mid-lower mantle pressures and at *T* = 1150 K. They found that the strengths of the cubic CaSiO_3_ perovskite at high temperatures are substantially weakened compared to that of the tetragonal phase at room temperature. In this study, we have used the experimentally measured strengths of lower-mantle minerals available at 1000 km in depth, corresponding to *P*_0_ = 38.6 GPa, and 1150 K as the reference state, from which the profiles of depth-dependent viscosity contrast between davemaoite and bridgmanite can be derived. We have considered two endmember deformation scenarios. First, the interconnected weak layer (IWL) deformation scenario assumes strain partitions in the weaker phase and stress is equally distributed between the phases, i.e., the uniform stress assumption. Second, the load-bearing framework deformation scenario assumes that rock deforms as a whole, and the strain rate is uniform, i.e., the uniform strain rate assumption. We set the homologous scaling factor *g* = 10 to 14 as suggested for MgSiO_3_ and MgO in previous studies (*49*). We find that our viscosity contrast profiles derived for a uniform temperature of *T* = 1150 K are in better agreement with the result of the IWL scenario (fig. S7). Therefore, we present the viscosity profile of IWL scenario in Fig. 4. We derived the viscosity profile under two mantle geotherms (i.e., cold and hot geotherms) (*50*, *51*). We find that the viscosities between the two minerals always differ by three to four orders of magnitude throughout the depth between 1000 and 1700 km, regardless of the choice of geotherm (Fig. 4). According to the IWL scenario, the strength of a rock mainly depends on the weaker phase (*49*); here, the weaker phase is always davemaoite. Therefore, increasing amounts of davemaoite in mantle rocks, for instance, by accumulation of subducted slabs or by fractional crystallization of the early magma ocean, may let the rock more prone to deformation when subjected to external forces. So, the ultrahigh melting temperatures of davemaoite at high pressures are not only potentially to change the crystallization sequence of magma ocean but also to change the rheological properties of solid mantle.

## DISCUSSION

Here, we report a theoretical study on the melting curve of davemaoite within its *P*–*T* stability field between 13 and 136 GPa. We find that our results are in very good agreement with the multi-anvil experiment measured at low pressures but substantially higher than diamond anvil cell experimental measurements at high pressures and other ab initio predictions. Our computational data indicate that davemaoite, as the third most abundant one, is much more difficult to melt than other minerals in the lower mantle. The majority of lower mantle is expected to be composed of bridgmanite, ferropericlase, and davemaoite. We already know that melting temperatures of the MgSiO_3_ endmember of bridgmanite are always lower than that of davemaoite. Increasing amounts of iron and aluminum accommodated in bridgmanite under compression will further lower its solidus by about 600 to 800 K (*52*). The MgO endmember of ferropericlase probably has a melting temperature ~200 K higher than that of davemaoite at the CMB pressure (*42*). However, ferropericlase is expected to be made of a solid solution containing a large fraction of iron, as ferrous iron preferentially partitions into ferropericlase under lower-mantle conditions (*25*). The formation of (Mg,Fe)O solid solution is able to lower the solidus of ferropericlase substantially (*48*). In addition, the spin transition of iron in ferropericlase will further lower its solidus (*53*). In contrast to bridgmanite and ferropericlase, the composition of davemaoite that appeared in super-deep diamond inclusions is remarkably clean, only with minor admixtures of Al_2_O_3_ and FeO (*54*). However, davemaoite tends to host higher content of large-ion incompatible elements than other minerals, including rare-earth elements, large-ion lithophile elements, and radioactive elements (K, U, and Th) (*27*–*29*). Considering that the concentrations of these incompatible elements in the lower mantle are far below the limit of saturation in the mineral, they should have a limited impact on the melting point of davemaoite. Other minor mineral phases in the lower mantle—such as post-perovskite bridgmanite, silica polymorphs, aluminous phases, and hydrous phases—are only regionally important. They often play subsidiary roles in the evolution of entire lower mantle and are unlikely to have higher–melting point than davemaoite based on available evidences from some of these phases (*4*, *55*). Overall, on the basis of our theoretical calculation, davemaoite is likely to be the most refractory mineral among major lower-mantle minerals.

An important factor that can modify the melting behavior of davemaoite is the mutual solubility of davemaoite and bridgmanite. In the past, the two minerals were basically regarded as independently present under lower-mantle conditions because of the distinct crystallographic symmetries of them (i.e., cubic for davemaoite versus orthorhombic for bridgmanite). Very recently, Ko *et al.* (*56*) found that at sufficiently high pressures (*P* > 40 GPa) and high temperatures (*T* > 2300 K), in pyrolite model, davemaoite can completely dissolve into bridgmanite and form a calcium-rich single perovskite. They reported that incorporation of iron and aluminum is a key to forming these solid solutions. They suggested a transition from a two-perovskite domain (TPD) to a single-perovskite domain (SPD) may occur between 1500 and 2200 km in depth depending on the mantle geotherm. Investigating whether and under what conditions TPD and SPD would exist obviously requires determining multicomponent phase diagrams including iron and aluminum impurities. Ab initio calculation remains extremely difficult to model these complex melting behaviors. Even if we accept that the above mentioned solid-solution scenario is capable of being held at some conditions, at cold mantle parts, davemaoite is still possibly present as the most refractory mineral.

The implication of the high melting temperature of davemaoite is critical for the evolution of the early magma ocean. An experimental study at 24 GPa found that the eutectic composition in the MgSiO_3_-CaSiO_3_ binary system was substantially shifted toward the MgSiO_3_ endmember (*14*). With the *T*_m_ exceeding 7000 K at the CMB pressure (if there was the CMB at that time, perhaps core and mantle might have not been separated yet), the eutectic of melting would keep moving toward the MgSiO_3_ endmember, resulting in the crystallization of the davemaoite out of the magma ocean. Because of the high *T*_m_, exceeding likely even the temperature in the center of Earth’s core, the solid layer of CaSiO_3_ would form an effective insulating layer on top of the heavy liquid mixture of iron and lighter elements, which is the liquid core. Such an insulating layer would preserve high temperature in the core, resulting in comparably late formation of the solid inner core. Nowadays, this layer might manifest itself as the low velocity zone adjacent to the CMB because propagation of the seismic signal in davemaoite is comparably slow (*30*, *31*).

To put the contemplation above on the solid quantitative ground, considerable computational work beyond the scope of this study is required. However, our paper provides clear path for such a work that eventually will explain the early evolution of Earth and the present state of the lower mantle. Besides, our study sets high standards in computing melting curves of Earth minerals. We believe that following these standards would result in removing most of the confusion in the field of computing melting curves.

## MATERIALS AND METHODS

### First-principles simulations based on DFT

Melting data in this study were mainly obtained by FPMD simulations on quantum mechanical level within the framework of DFT. Our FPMD calculations were performed using the Vienna Ab initio Simulation Package, a proprietary atomic-scale materials modeling software from first principles (*57*). Electronic structure calculations were performed by plane wave basis sets with an energy cutoff of 520 eV. Interactions between electrons and ions were described by the projector-augmented wave method (*58*). Different types of exchange-correlation energy functional approximations were tested in this study, including the local density approximation (LDA) (*59*) and generalized gradient approximations implemented by the form of PBE (*41*) and PBEsol (*37*). Our choice of the configuration of valence electrons for each element coincides with previous computational studies (*11*). All convergence tests have been performed. We used a 0.5-fs timestep for the FPMD simulations. We have approximated the CaSiO_3_ perovskite structure by the supercell with at least 135 atoms (3 × 3 × 3 unit cells) (e.g., in the Z method) and at most 1080 atoms (6 × 6 × 6 unit cells) (e.g., in the two-phase method). The FPMD runs were performed for the gamma point in the Brillouin zone only. At the size of the supercell, this is quite sufficient. The finite temperatures for the electronic structure and force calculations were implemented within the Fermi-Dirac smearing approach.

### Z method calculations

Determination of the equilibrium melting point from computer simulation is not as straightforward as one think because a strong hysteresis effect exists in the first-order phase transition (*60*). This effect can cause substantial overheating and obtain a much higher temperature than that of the true value. Here, we used the Z method (*11*, *35*) to avoid the overheating problem. In the Z method, the cell consists of a defect-free solid as the initial configuration. Then, the FPMD run is performed in the NVE ensemble at a number of different total energies. The total energy of each run is assigned by setting the initial velocities of atoms drawn from a Maxwell distribution. After the total energy exceeds a critical value, the cell spontaneously melts. The temperature of the solid will drop down to a lower value corresponding to the temperature of the melted liquid. When the temperature of solid approaches the limit of superheating, the time to melt (i.e., waiting time) will become infinitely long. An infinite simulation is impractical. To avoid this problem, we adopted the waiting time analysis procedure similarly used by Braithwaite and Stixrude (*11*). The maximum length of each NVE run was set to 30 ps. The equilibrium melting temperature was determined by extrapolating the waiting time to infinite value by fitting data to an empirical relation between waiting time and temperature of liquid (fig. S1) (*61*).

### Derivation of melting curve from Gibbs thermodynamic surface

Two approaches can be used to derive the melting curve from a known point on the curve, the integration of Clausius-Clapeyron equation and the approach of constructing Gibbs thermodynamic surface. Both approaches require performing a large number of NVT (*N*, number of atoms; *V*, volume; *T*, temperature) ensemble FPMD runs on the solid and liquid states of material. In this study, we adopted the latter approach to derive the melting curve because the second approach can provide more information than the first one (such as to determine the limit of superheating and the interval of coexistent states) and then notably reduce the number of trial calculations in the Z method and two-phase method (*34*). The so-called Gibbs thermodynamic surface is a three-dimensional geometrical surface in the space of three coordinates, volume (*V*), entropy (*S*), and total energy (*E*). It was first proposed by J. W. Gibbs to discuss the conditions of thermodynamic equilibrium and stability for a compositional invariable system. To construct this surface from NVT ensemble simulation results, we have assumed that heat capacity and Grüneisen parameter at constant volumes are both invariable quantities, which is a reasonable assumption for silicate solids and liquids at high temperatures around the melting phase transition. Following our previous work on model system (*34*), we derived the equations of *E*(*V*) and *E*(*S*) at constant entropy and constant volume, respectively. The Gibbs thermodynamic surface was built by interpolating data between a grid of volume and entropy. The melting point was determined by the definition, i.e., the equivalence of Gibbs free energies of solid and liquid phases at a given pressure. The melting curve was fitted by a number of melting points at different pressures. To confirm the validity of our approach, we have tried the integration of Clausius-Clapeyron approach and obtained indistinguishable melting curves with respect to our approach. We have applied a constant pressure correction in this approach for different exchange-correlation functionals to account for the systematic pressure shift in DFT. The correction pressure can be computed by $P_{c}=-P_{\mathrm{DFT}}(V_{\mathrm{ref}}^{0})$, where *P*_DFT_(*V*) is the function of third-order Birch-Murnaghan equation of state calculated by DFT and $V_{\mathrm{ref}}^{0}$ is the reference volume at ambient conditions. Here, the experimental volume of unit cell of davemaoite at zero pressure 45.58 Å^3^ is used as the reference volume (*62*). The pressure correction *P*_c_ is −7.22 GPa for PBE functional and 5.19 GPa for LDA functional. The equation of state calculated from PBEsol functional is in good agreement with experiment without applying pressure correction (fig. S3).

### Two-phase method calculations

In the two-phase method (*43*), the cell consists of liquid and solid parts with a common interface. Then, the run is performed in the NPT (*N*, number of atoms; *P*, pressure; *T*, temperature) ensemble until the cell becomes homogeneous, either solid or liquid. If it is liquid, then the P-T point is above the melting curve; if it is solid, then the P-T point is under the melting curve. In practice, one does not have to run it to completely homogeneous state—the trend becomes pretty obvious by simply looking at the change of the RDF. The two-phase cell was obtained by freezing half of the thermally equilibrated atom CaSiO_3_ perovskite cell and running NVT ensemble simulations using machine learning potential at very high temperatures to melt the other half. In the end, we obtain half solid and half liquid atoms with a common interface. Then, at a fixed given pressure, we ran several two-phase runs at a number of temperatures until the temperature gap between the homogeneous solid and the homogeneous liquid states was sufficiently narrow. Machine learning interatomic potential was used to facilitate the two-phase method calculation and to verify the size and time effect. We have built the MLFF using the on-the-fly active learning approach (*63*). The training data were generated by performing ab initio two-phase simulations on the supercell of 270 atoms (6 × 3 × 3 unit cells) under NPT ensemble. The MLFF-based simulation was run for 100 ps at 136 GPa and 7500 K. A movie was made for this simulation by OVITO software (*64*). It shows that the two-phase system mostly crystallized at this pressure and temperature.

### Thermodynamic integration calculations

The thermodynamic integration method (*39*) calculations were generally followed the same approach used by Hernandez *et al.* (*13*). We performed a direct thermodynamic integration from the ideal gas reference system (λ = 0) to the fully interacting liquid silicate system (λ = 1) to obtain the Helmholtz free energy of liquid phase. To address the issue that the integrand becomes too large at a small λ value, we transformed the variable from λ to *x* using the following relation: λ(*x*) = [(*x* + 1)/2]^1/(1−*k*)^. The numerical integration was performed by an eight-point Gauss-Lobatto quadrature with *k* = 0.8. The FPMD runs for thermodynamic integration were carried out in the NVT ensemble.
