# Supplementary material for: Davemaoite as the mantle mineral with the highest melting temperature
Source: Sci Adv. 2023 Dec 6;9(49):eadj2660. doi: 10.1126/sciadv.adj2660 (PMC10699773; doi:10.1126/sciadv.adj2660)
Supplement: Supplementary file 1 — Figs. S1 to S7 Tables S1 to S3 Legend for movie S1 [file sciadv.adj2660_sm.pdf]

Science Advances
AAAS

# Supplementary Materials for

## Davemaoite as the mantle mineral with the highest melting temperature

Kun Yin *et al.*

Corresponding author: Kun Yin, yinkun@cdut.edu.cn; Xiancai Lu, xcljun@nju.edu.cn

*Sci. Adv.* **9**, eadj2660 (2023)

DOI: 10.1126/sciadv.adj2660

**The PDF file includes:**

Figs. S1 to S7
Tables S1 to S3
Legend for movie S1

**Other Supplementary Material for this manuscript includes the following:**

Movie S1

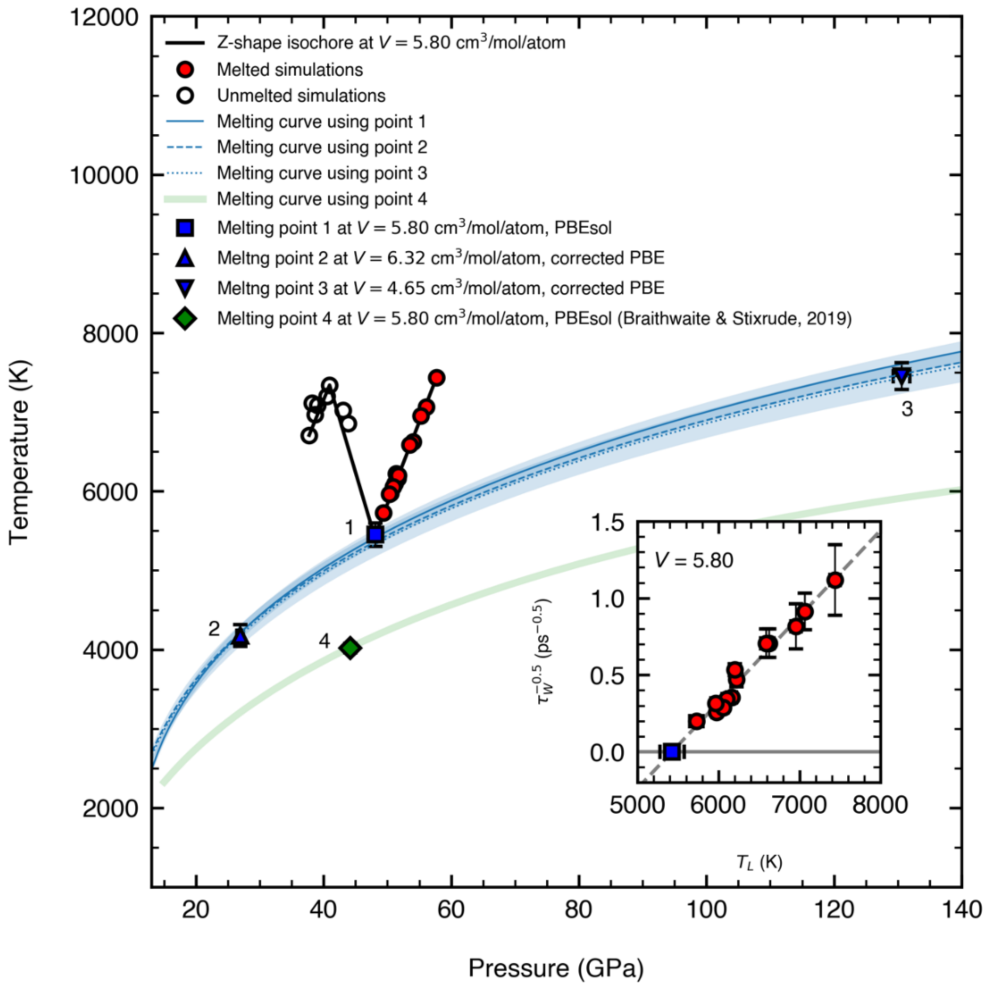


**fig. S1. Results of Z method simulation for davemaoite.** Simulations were performed at three constant volumes ($V$ = 4.65, 5.80 and 6.32 cm$^3$/mol/atom) using NVE ensemble and PBE or PBEsol functional. Each NVE-ensemble FPMD simulation was run with a maximum length of 30 ps. Some runs melted before the end of simulation and others remained solid within the maximum simulation time. For the melted simulations (red points), temperatures of the liquid states ($T_L$) and the waiting time to completion of melting ($\tau_w$) were used for the waiting-time-analysis procedure. In this procedure, $T_L$ and $\tau_w$ were fitted to the following empirical relation: $\tau_W^{-0.5} = A(T_L - T_M)$. Finally, the equilibrium melting point ($T_m$) was derived by the intercept of the above relation at $\tau_W^{-0.5} = 0$ (the inset figure). Data of melting points 1 to 4 are given in Table S1. Melting point obtained by Braithwaite and Stixrude (*11*) was show for comparison. They obtained the melting point using the similar method at the same constant volume, but with a larger timestep. Pressure correction has been applied to PBE.

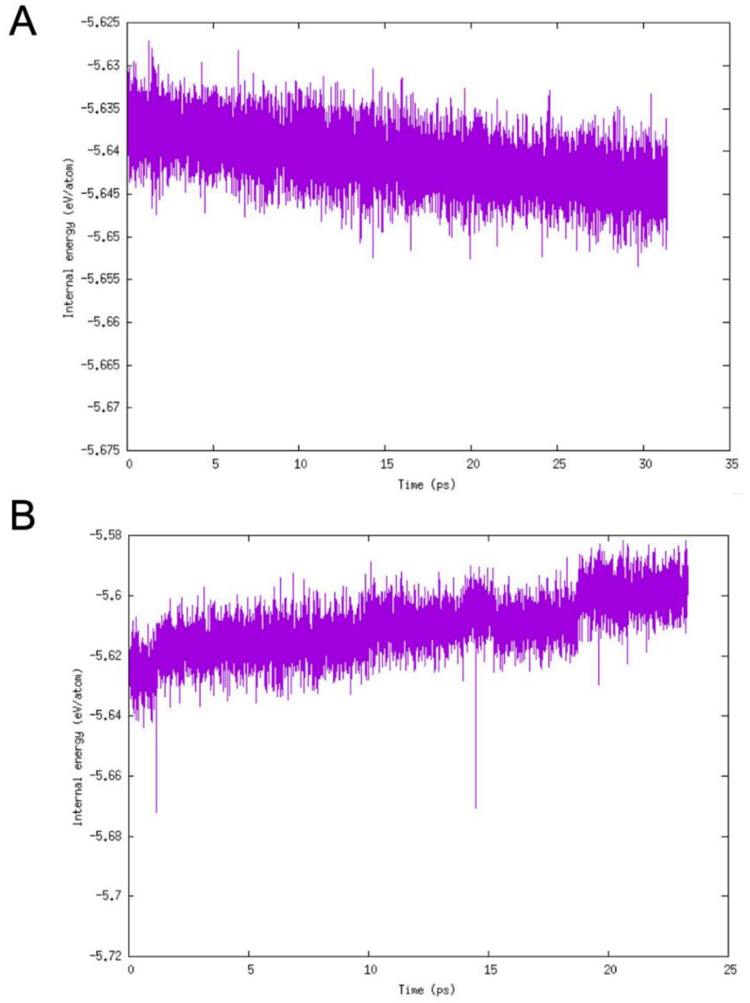


**fig. S2. Trajectory of the internal energy of a microcanonical ensemble simulation of davemaoite.** (**A**) timestep 0.5 fs; (**B**) timestep 1 fs. The constant total internal energy of the simulation was controlled by setting the initial velocities of atoms drawn from a Maxwell distribution. The maximum length of microcanonical (NVE) run is about 32 ps and 23 ps for simulation with a timestep of 0.5 fs and 1 fs, respectivley. A 3 × 3 × 3 supercell of cubic $CaSiO_3$ perovskite with five atoms in the unit cell is used for building the simulation cell. It can be observed that the internal energy is not perfectly conserved in the whole simulation period, but slowly drifts away from the initial value with an average amount of 0.0002 and 0.0015 eV/atom/ps for 0.5 fs and 1 fs cases, respectively.

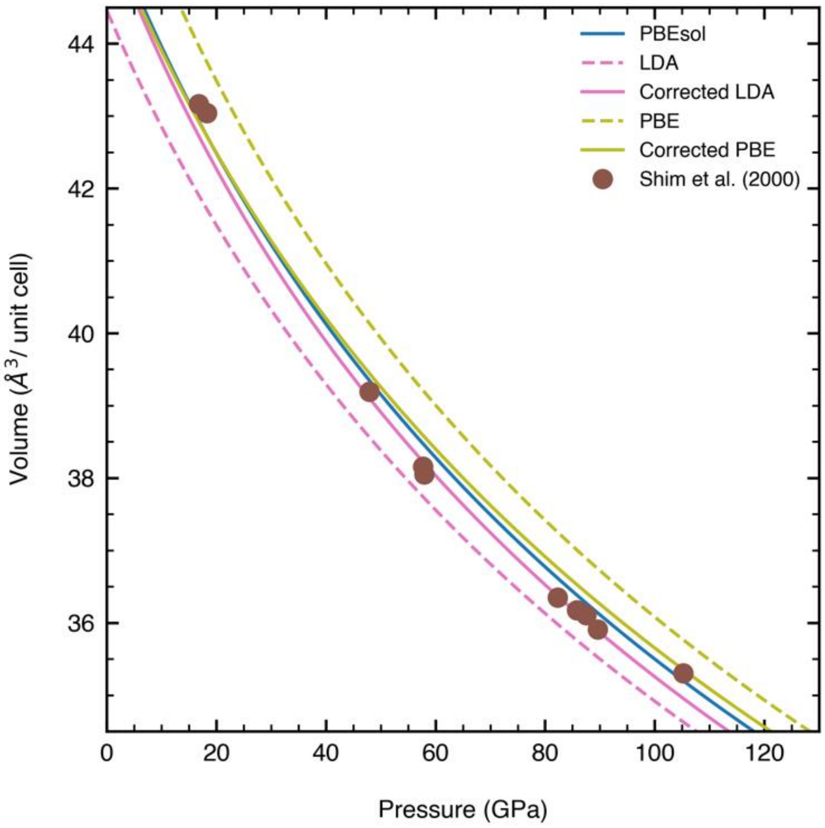


**fig. S3. Static equation of state of davemaoite with and without pressure correction.** DFT volume-pressure data obtained at zero-temperature were fitted to the third-order Birch-Murnaghan equation of state for different exchange-correlation functionals. LDA and PBE functionals were corrected by the zero-pressure experimental volume. The corrected curves for LDA and PBE functionals are equivalent to shifting the uncorrected curves by a constant volume. This simple correction can greatly improve the under- or over-estimation of volume by LDA or PBE, respectively. PBEsol functional without applied pressure correction was already in good agreement with the experimental data of Shim *et al.* (*62*).

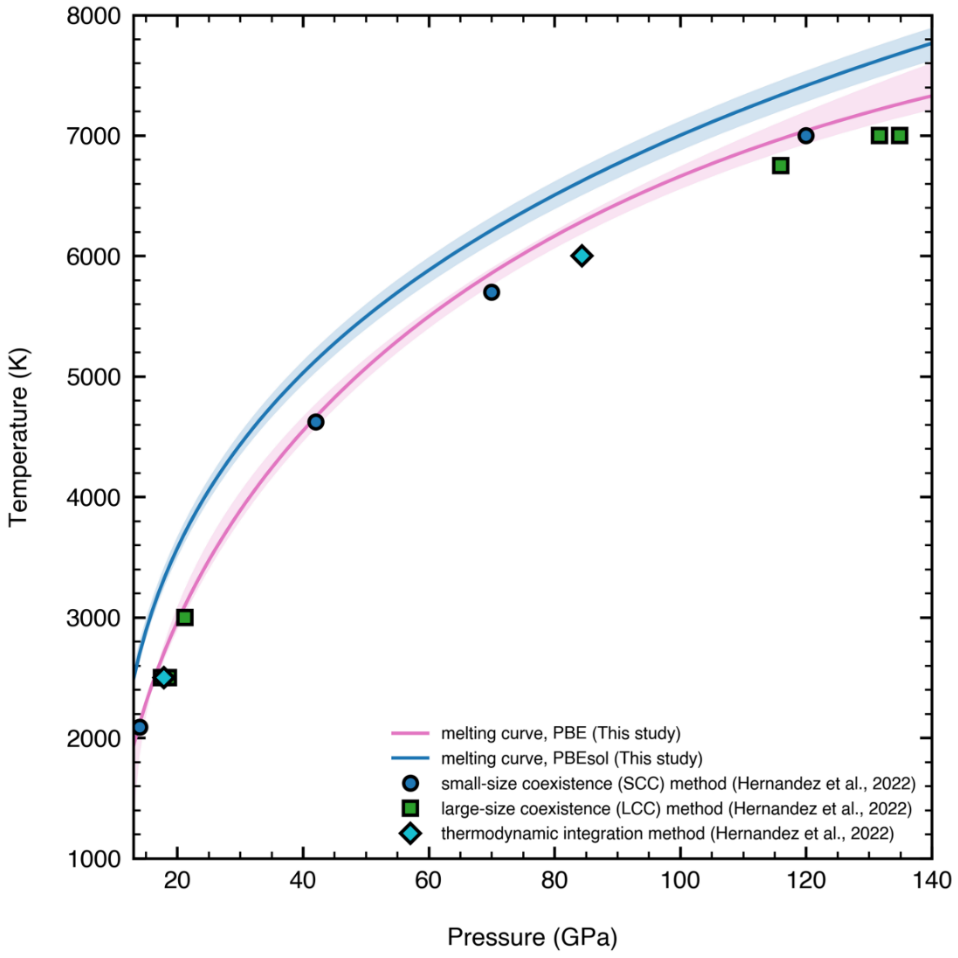


**fig. S4. Melting curve of davemoaite derived from PBE functional and PBEsol functional.** Our computed melting curve is compared with previous calculations by Hernandez *et al.* (*13*) who used three different methods, i.e., the small-size coexistence (SCC), large-cell coexistence (LCC) and thermodynamic integration methods. The melting curves were determined by raw DFT data without applying pressure correction to functional.

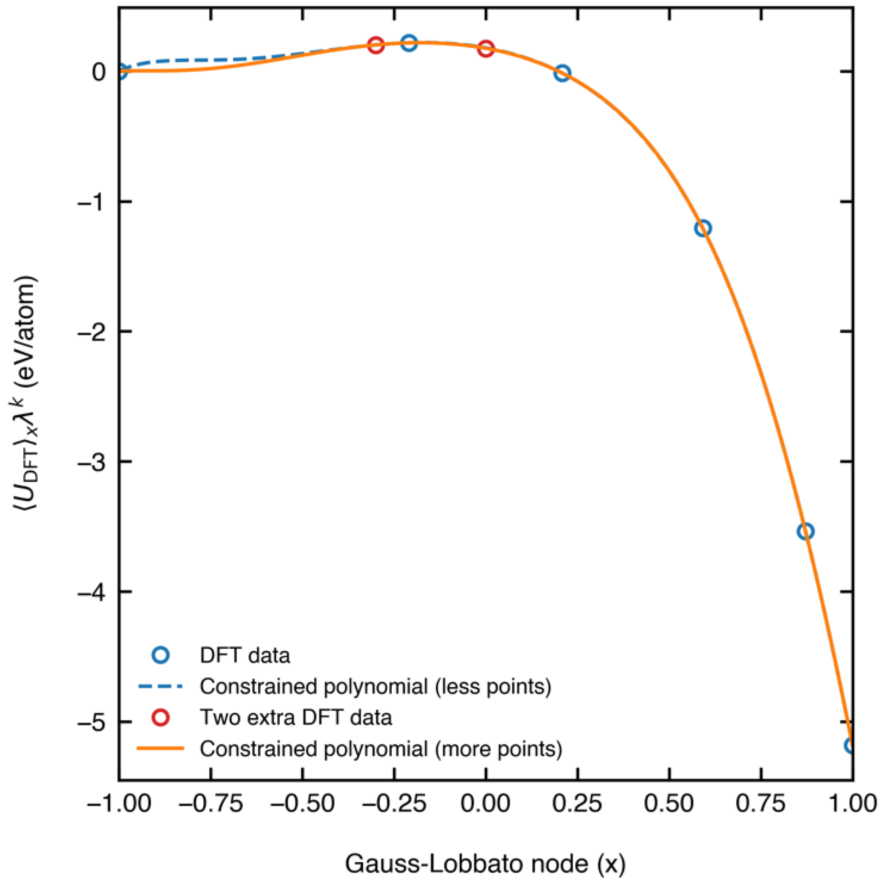


**fig. S5. Potential energy differences between the ideal gas and DFT liquid $CaSiO_3$ as a function of the coupling parameter $\boldsymbol{\lambda}$ in thermodynamic integration.** $\boldsymbol{\lambda}$ has been transformed to Gauss-Lobatto node *x* by integrand transformation: $\boldsymbol{\lambda(x) = ((x+1)/2)^{1/(1-k)}}$ and $k = 0.8$. The DFT data were obtained using NVT ensemble FPMD simulations performed at $T = 6{,}000$ K and $V = 7.352$ Å$^3$/atom. Polynomial fits were constrained to have a zero slope at $x = -0.209299218$. Fits using different number of DFT data were tested and shown as solid and dashed lines. Using more points to fit apparently change the shape of polynomial curve at low $\boldsymbol{\lambda}$ values, hence will change the integral of potential energies.

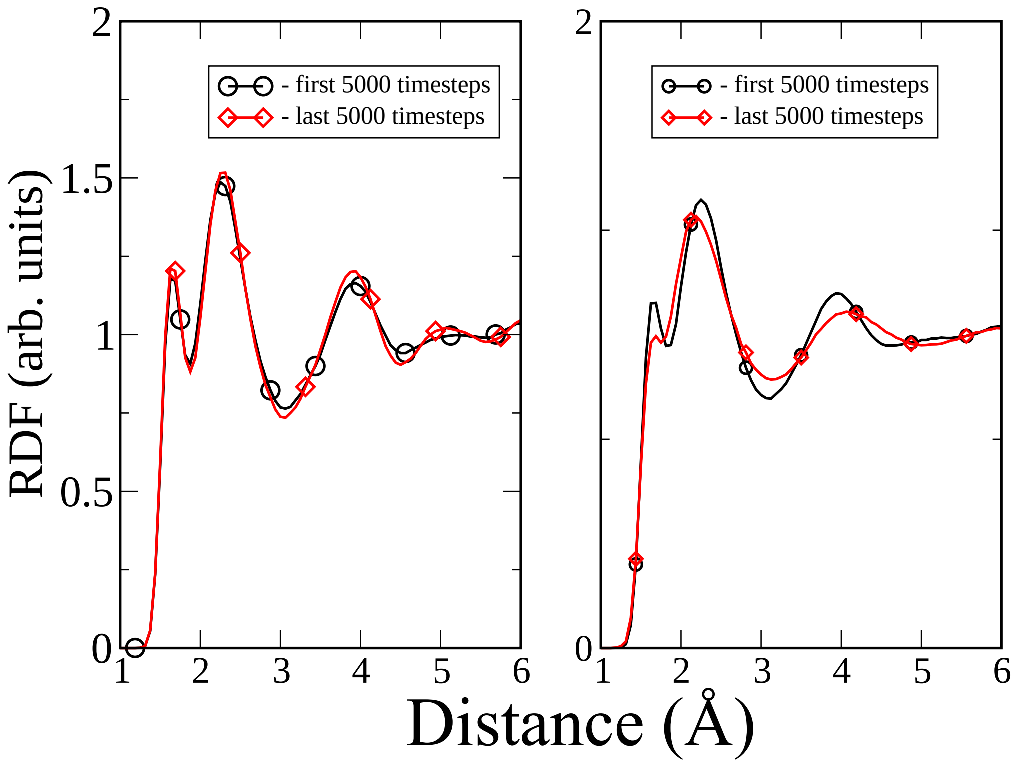


**fig. S6. Total radial distribution functions (RDF) at *T* = 7,000 K (left) and *T* = 8,000 K (right) at the *P* = 150 GPa.** The black curves (marked with circles) were calculated as average over the timesteps between 1 and 5,000. The red curves (marked with diamonds) were calculated over the last 5000 timesteps of corresponding runs, that is between 45,000th and 50,000th timesteps (left) and 15,000th and 20,000th timesteps (right). The ordering at the 7,000 K looks pretty modest, perhaps because the major change happens already at the first 5,000 timesteps. The disordering (melting) at 8,000 K leads to the disappearance of the first peak, which is a clear sign of melting.

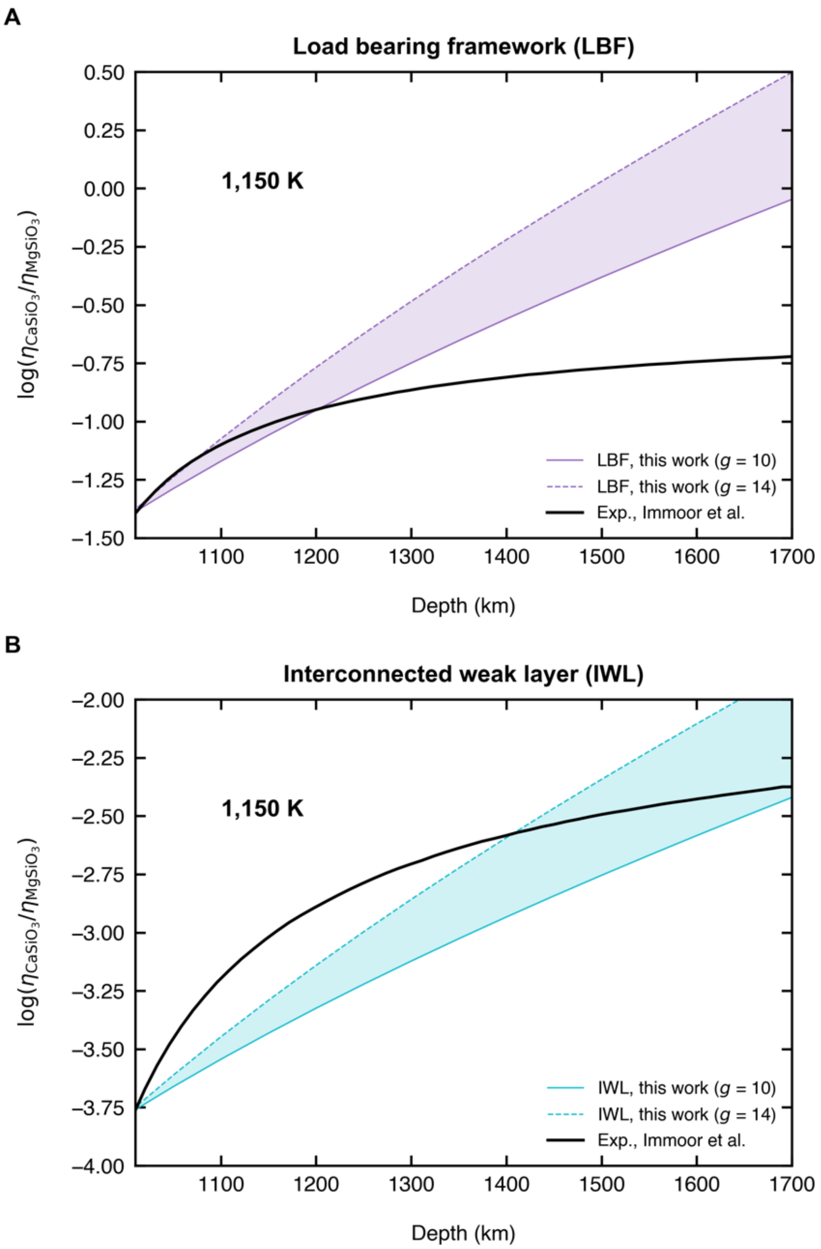


**fig. S7. Viscosity contrast profiles between $CaSiO_3$ and $MgSiO_3$ at uniform temperature.** Two deformation scenarios, i.e., (**A**) load-bearing framework (LBF) scenario, and (**B**) interconnected weak layer (IWL) scenario, were considered. Data were derived from the reference state measured at a uniform temperature of 1,150 K and the pressure corresponding to 1,000 km depth. Immoor *et al.* (*33*) have modeled the viscosity contrast between lower-mantle phases under the same P-T conditions based on experimental measurement. Our calculations are more consistent with the IWL scenario modeled by experiment.

**table S1. Data of melting point obtained by the Z method calculations.** Previous calculation by Braithwaite and Stixrude (BS19) (*11*) is listed for comparison. The melting points have been corrected by considering the effect of using different exchange-correlation functionals, PBE or PBEsol.

| **Point No.** | $P$ (GPa) | $T$ (K) | $V$ (Å$^3$/atom) | $V$ (cm$^3$/mol/atom) | **Functional** | **Reference** |
|---|---|---|---|---|---|---|
| 3 | 130.6 ± 1.3 | 7454 ± 171 | 7.722 | 4.65 | PBE (pressure corrected) | This study |
| 2 | 26.9 ± 0.5 | 4176 ± 138 | 10.50 | 6.32 | PBE (pressure corrected) | This study |
| 1 | 48.1 ± 1.0 | 5451 ± 150 | 9.631 | 5.80 | PBEsol | This study |
| 4 | 44.2 ± 0.2 | 4020 ± 60 | 9.631 | 5.80 | PBEsol | BS19 |

**table S2. Parameters of the Kechin equation that constrain the upper and lower bounds of the melting curves of $MgSiO_3$ and $CaSiO_3$.** During curve fitting, $T_0$ was fixed. The upper and lower bounds of melting curve represent the uncertainties of the melting temperature.

| Phase | Bound of melting curve | $T_0$ (K) | $P_0$ (GPa) | $a$ (GPa) | $b$ | $c$ (GPa) |
|---|---|---|---|---|---|---|
| $MgSiO_3$ | Upper | 2900 | 21.0 | 13.7 | 0.354 | 1279 |
| | Lower | 2900 | 24.1 | 18.0 | 0.379 | 861.9 |
| $CaSiO_3$ | Upper | 4020 | 23.5 | 14.0 | 0.321 | 2790 |
| | Lower | 4020 | 25.6 | 15.9 | 0.327 | 2380 |

**table S3. Results of thermodynamic integration calculations for liquid $CaSiO_3$.** We calculated Helmholtz free energy of the liquid phase by: $\boldsymbol{F} = \boldsymbol{F}_{\text{IG}} + \Delta\boldsymbol{F}$. $\boldsymbol{F}_{\text{IG}}$ is the contribution of a non-interacting ideal gas reference system, $\Delta\boldsymbol{F}$ is the free energy difference between the ideal gas reference system and the fully interacting liquid system. $\Delta\boldsymbol{F}$ was calculated by the thermodynamic integration method with the following formula: $\Delta\boldsymbol{F} = \int_0^1 \langle \boldsymbol{U}_{\text{DFT}} \rangle_\lambda \boldsymbol{d\lambda} = \frac{1}{2(1-k)} \int_{-1}^{1} \langle \boldsymbol{U}_{\text{DFT}} \rangle_x \boldsymbol{\lambda^k dx}$, where $\langle \boldsymbol{U}_{\text{DFT}} \rangle_\lambda$ is the potential energy of DFT calculation carried out in the NVT ensemble with a Nosé-Hoover or Langevin thermostat at a given coupling parameter $\boldsymbol{\lambda}$. To avoid the integrand becomes too large, we transformed $\boldsymbol{\lambda}$ to *x* with the following relation: $\boldsymbol{\lambda(x) = ((x+1)/2)^{1/(1-k)}}$. The numerical integration was performed by an 8-point Gauss-Lobatto quadrature and $k = 0.8$. Simulations with $\boldsymbol{\lambda}$ smaller than 0.005252 failed, as the atoms come too close to each other for the electronic wave function to converge. A constrained polynomial was fitted to $\langle \boldsymbol{U}_{\text{DFT}} \rangle_x \boldsymbol{\lambda^k}$ data points prior to integration over *x*.

| $N = 135$, $V = 7.352$ Å$^3$/atom, $T = 6000$ K | | | |
|---|---|---|---|
| $x$ | $\lambda$ | $\langle U_{\text{DFT}} \rangle_x$ (eV/atom) (This study) | $\langle U_{\text{DFT}} \rangle_x$ (eV/atom) (Hernandez *et al.*, 2022) (*13*) |
| -0.3 | 0.005252 | 13.4(1) | - |
| -0.209299218 | 0.009659 | 8.9(1) | 9.0 |
| 0 | 0.031250 | 2.8(1) | - |
| 0.209299218 | 0.080820 | -0.12(5) | -0.11 |
| 0.591700181 | 0.319269 | -3.01(1) | -3.02 |
| 0.871740149 | 0.717923 | -4.61(2) | -4.61 |
| 1 | 1 | -5.18(1) | -5.17 |
| Free energies | | | |
| | $P$ (GPa) | 151.6(2) | 152 |
| | $F_{\text{IG}}$ (eV/atom) | -6.73 | -6.73 |
| | $\Delta F$ (eV/atom) | -3.08 | -3.06 |

**movie S1. Simulation results of the 2-phase method of davemaoite.** This simulation was performed by machine learning force field (MLFF)-based molecular dynamics (MD) under NPT ensemble ($N$ = 1,080 atoms, $P$ = 136 GPa and $T$ = 7,500 K). This simulation was run for 100 ps. Snapshot of atomic configuration in the (010) plane was taken every 100 steps. Ca, Si, O atoms are represented as copper, cyan, and red balls, respectively. The total movie is about 42 seconds. We will see that after 12 seconds in the movie (corresponding to about 30 ps in the MD simulation) the 2-phase system has been mostly crystallized. This animated movie was created with OVITO software (https://ovito.org) (*64*).
